# Dizziness and slow heart rate during exercise

**DOI:** 10.1007/s12471-017-0983-2

**Published:** 2017-04-11

**Authors:** R. Joustra, M. Boulaksil, H. W. Meijburg, J. L. Smeets

**Affiliations:** 1 Department of Cardiology, Jeroen Bosch Hospital, ’s-Hertogenbosch, The Netherlands; 2 Department of Cardiology, Radboud University Medical Center, Nijmegen, The Netherlands

A 75-year-old woman was presented to our emergency department because her pulse had become slow during physiotherapy exercises. She had a history of bypass surgery for severe triple vessel disease and severe left ventricular dysfunction. Furthermore, one month before presentation, she had been hospitalised with progressive heart failure presumed to have been caused by a high ventricular rate during an episode of atrial fibrillation. In order to achieve rate control, she was treated with digoxin and bisoprolol. She recovered well from this episode of decompensated heart failure.

On presentation she had a two-day history of dizziness and light-headedness. On clinical examination her pulse rate was 30 bpm and blood pressure was 160/45 mm Hg. There were no clinical signs of heart failure or dehydration. The electrolyte panel was normal. However, her renal function worsened to an estimated MDRD-GFR of 22 ml/min compared with 37 ml/min one month earlier. The ECG at presentation is shown in Fig. 1.

**Fig. 1:**
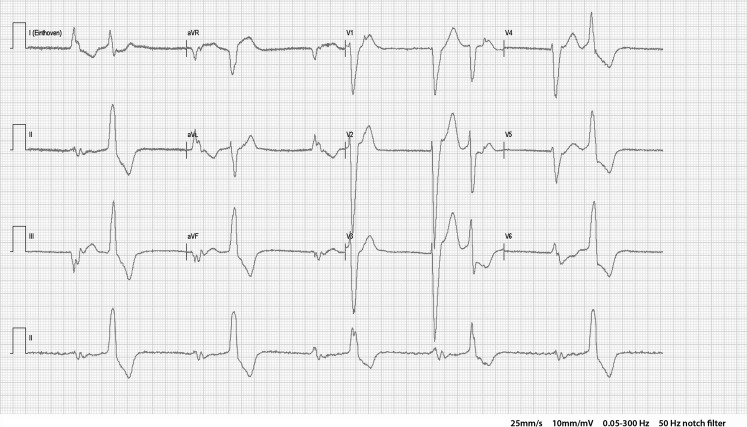
ECG at presentation

1. What is your electrocardiographic diagnosis?
2. What is the most likely mechanism?

## Answer

You will find the answer elsewhere in this issue.

